# The genetic basis of discrete and quantitative colour variation in the polymorphic lizard, *Ctenophorus decresii*

**DOI:** 10.1186/s12862-016-0757-2

**Published:** 2016-09-06

**Authors:** Katrina J. Rankin, Claire A. McLean, Darrell J. Kemp, Devi Stuart-Fox

**Affiliations:** 1 School of BioSciences, The University of Melbourne, Parkville, VIC 3010 Australia; 2 Department of Sciences, Museum Victoria, Carlton Gardens, VIC 3053 Australia; 3 Department of Biological Sciences, Macquarie University, North Ryde, NSW 2109 Australia

**Keywords:** Colour polymorphism, Heritability, Mendelian, Animal model, Quantitative trait, Testosterone, Microsatellite, Image analysis

## Abstract

**Background:**

Colour polymorphic species provide invaluable insight into processes that generate and maintain intra-specific variation. Despite an increasing understanding of the genetic basis of discrete morphs, sources of colour variation within morphs remain poorly understood. Here we use the polymorphic tawny dragon lizard *Ctenophorus decresii* to test simple Mendelian models for the inheritance of discrete morphs, and to investigate the genetic basis of continuous variation among individuals across morphs. Males of this species express either orange, yellow, orange surrounded by yellow, or grey throats. Although four discrete morphs are recognised, the extent of orange and yellow varies greatly. We artificially elevated testosterone in F0 females and F1 juveniles to induce them to express the male throat colour polymorphism, and quantified colour variation across the pedigree.

**Results:**

Inheritance of discrete morphs in *C. decresii* best fit a model whereby two autosomal loci with complete dominance respectively determine the presence of orange and yellow. However, a single locus model with three co-dominant alleles for orange, yellow and grey could not be definitively rejected. Additionally, quantitative expression of the proportion of orange and yellow on the throat was strongly heritable (orange: h^2^ = 0.84 ± 0.14; yellow: h^2^ = 0.67 ± 0.19), with some evidence for covariance between the two.

**Conclusions:**

Our study supports the theoretical prediction that polymorphism should be governed by few genes of major effect, but implies broader genetic influence on variation in constituent morph traits.

**Electronic supplementary material:**

The online version of this article (doi:10.1186/s12862-016-0757-2) contains supplementary material, which is available to authorized users.

## Background

Colour polymorphic species are often used as model systems in evolutionary biology because they offer obvious visual markers of genetic variation (e.g. [1–3]). True colour polymorphism refers to the presence of multiple discrete and genetically-determined morphs within an interbreeding population, with the rarest too frequent to arise from recurrent mutation [4, 5]. In species for which the genetic basis of colour polymorphism has been studied, alternative morphs are often explainable by the simple Mendelian segregation of few alleles across limited loci (reviewed in [6, 7]). For example, Lank et al’s classic study on ruffs (*Philomachus pugnax*) showed that male colouration/reproductive strategy is primarily controlled by a single autosomal locus with two alleles [8], see also [9]. Similarly, colour patterns in the Coqui frog (*Eleutherodactylus coqui*) are consistent with the segregation of five autosomes at a single locus: In this case all alleles code for striped patterns, and exhibit co-dominant effects on phenotypes, except for one recessive allele that produces un-striped morphs in homozygotes [10]. In the side blotched lizard, *Uta stansburiana,* males express a colour polymorphism again in association with reproductive strategy that appears to be controlled by a single autosomal locus with three co-dominant alleles *o, b, y* (orange, blue and yellow, respectively; [11, 12]). Such studies attest to how divergent phenotypes may result from somewhat simple regulatory mechanisms, as predicted by broader theory for polymorphism [13].

In colour-polymorphic species, alternative colour phenotypes are often associated with differences in morphology, behaviour, physiology and/or life-history [7, 13, 14]. Theory proposes that morph-specific trait combinations represent alternative peaks across the fitness landscape [15]. These trait combinations may be driven by pleiotropy, where a single gene influences multiple phenotypic traits. For example, genes involved in pigment production may also act on other tissue types [16–18] and/or the presence of a given morph within a population may influence social interactions and consequently morph fitness (e.g. [19]). Alternatively, or in addition, selection may favour phenotypes with particular trait combinations. Such correlational selection will result in the formation of linkage disequilibrium at loci governing morph-specific traits, which will be sustained in-turn by processes such as frequency-dependent selection [13, 20]. Selection of this nature is therefore expected to act upon genetic architecture (i.e., the G-matrix) to favour mechanisms of broad phenotypic effect [13]. This may be most likely when genes underlying trait combinations reside in regions of major effect, mediated via loci in close physical proximity and/or modifier loci which regulate multiple genes. The presence of such mechanisms, conceptualised as “supergenes” [21], is consistent with observations in exemplar systems such as ruffs [8] and side-blotched lizards [12, 14, 22]. Emerging evidence has provided further support across a range of systems, and identified mechanistic bases in gene regulation (e.g. Lake Malawi cichlids [23]), reviewed in ([24], *Heliconius* butterflies [25], reviewed in [26]) and chromosomal inversion (e.g. ruffs [9], white-throated sparrows [27]).

Although discrete colour morphs are expected to be governed by few genes of major effect, colour expression may vary substantially and/or continuously within morphs. For example, in ruffs, territorial-versus-satellite males have predominantly dark-versus-light ornamental plumage, but colouration within each category appears ‘hypervariable’ [9]. Within-morph variation of this nature is not easily explained by the segregation of alleles across one or two loci. Moreover, little is known about the relative contribution of polygenic, environmental and interactive effects upon such variation. Present insight derives largely from intensively-studied systems. In guppies (*Poecilia reticulata*), for example, variation among males in the size of different ornamental colour traits (e.g., orange, black & iridescent markings) is tightly controlled by Y-linked genes, yet more subtle variation such as the chroma of orange spots shows strong sensitivity to the environment [28]. Likewise, the contribution of phenotypic plasticity has been explored for polymorphic systems such as *Hypolimnas* butterflies [29]. However, despite enduring interest in the genetic regulation of colour polymorphism (reviewed in [6, 7, 9, 30]), few studies have explicitly addressed the genetic basis of quantitative variation within discretely-classified morphs (but see [31, 32] for notable exceptions in *Heliconius*).

**Fig. 1:**
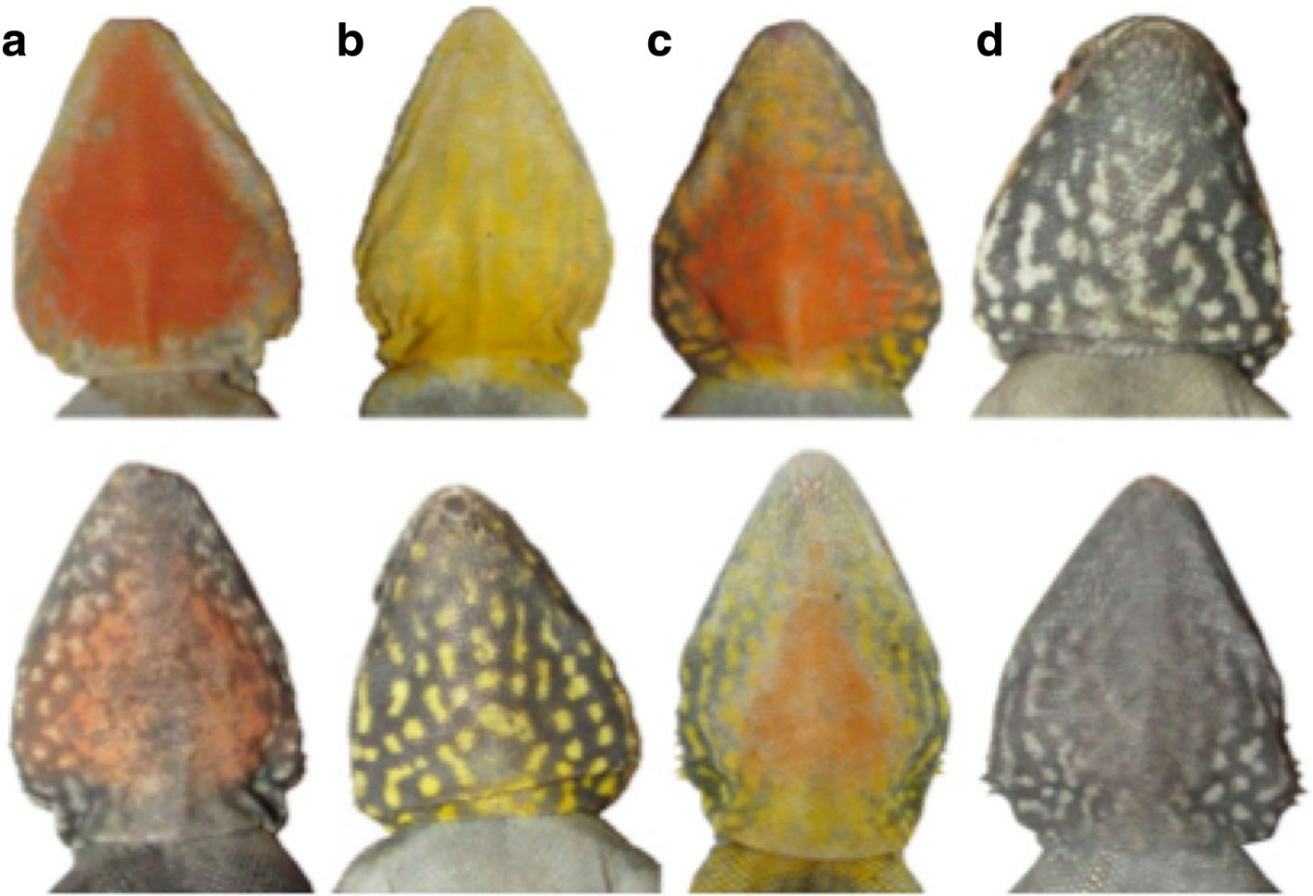
Four morph types, exemplifying within-morph variability (top and bottom row of images). The proportion of colour and degree of reticulations varies between individuals. Orange (**a**) and Yellow (**b**) morph have only orange or yellow respectively on their throat; Orange + Yellow (**c**) has an orange central patch surrounded by yellow; Grey morph (**d**) lacks either orange or yellow colouration

The tawny dragon, *Ctenophorus decresii*, is a small, sexually dimorphic agamid lizard (mean snout-vent length 80 mm and 70 mm for males and females, respectively), comprising two genetically distinct lineages, both endemic to South Australia [33, 34]. Males of the southern lineage are monomorphic for throat colour, while those from the northern lineage express one of four discrete throat colours (orange, yellow, grey or orange + yellow) at sexual maturity (approx. 18 months), which persists throughout their life [33, 35–37]. Orange and yellow morph males have only orange or yellow on their throat respectively; orange + yellow morphs have an orange central patch surrounded by yellow; and the grey morph lacks any orange or yellow colouration [37]. Within each morph category, the size of the colour patch and patterning varies substantially among individuals (Fig. 1; [34, 37, 38]). The colour morphs in Northern *C. decresii* correspond to different behavioural strategies [39]. Orange males are most aggressive, and grey least aggressive. Orange + yellow males behave similarly to yellow males, with aggression conditional on the intruder’s throat colour: orange and yellow males are challenged more aggressively than grey males. In addition to being least aggressive, the grey morph also exhibits lower boldness towards a simulated predator than the other three morphs [39]. Although the morphs differ in behaviour and associated endocrine levels (Yewers, Jessop, Pryke, Stuart-Fox, unpublished data), there are no apparent differences in morphology, habitat preference or other traits which might otherwise affect colour expression [37, 39]. Thus, as in other species with sex-limited colour polymorphism, colour forms are associated with differences in behaviour and correlated traits.

In this study, we investigate the genetic basis of throat colouration in the polymorphic northern lineage of *C. decresii* using captive-bred offspring from wild-caught parents over two breeding seasons. As with many colour polymorphic species, the tawny dragon is sexually dichromatic. Males express one of the four throat colours at sexual maturity, whereas females exhibit a cream coloured throat, sometimes with a flush of yellow and/or a yellow bib [37]. Juveniles of both sexes (<18 months of age) exhibit cream throats and the cryptic mottled brown dorsal colouration typical of mature females. Importantly, however, all four discrete male morphs can be expressed in females and juveniles using a simple testosterone treatment (see also [40]). We applied this treatment to a parent-offspring pedigree to investigate the genetic basis of both qualitative and quantitative colour variation. Our aims were (a) to assess whether/how variation at the level of discrete morph is accounted by alternative models based on simple Mendelian inheritance, and (b) to estimate the quantitative genetic basis of variation in key features (orange and yellow) within and between morphs.

## Methods

### Animal housing and husbandry

Totals of 51 male and 20 female *C. decresii* were captured from rocky outcrops off reserves in the vicinity of Warren Gorge, Flinders Ranges, South Australia (31.4222° S, 138.7050° E), in October and November 2011 and subsequently housed in the animal facility at The University of Melbourne, Australia. Three of the females produced 10 offspring (5 females, 5 males) in captivity from unknown wild fathers in December 2011. These offspring had reached sexual maturity prior to breeding experiments, giving a total of 56 male and 25 female adults in the captive population. Lizards were housed individually in 55 L x 34 W x 38 D cm opaque plastic tubs containing a layer of sand, and provided with a ceramic tile hide for shelter. The room was maintained at temperatures and lighting regimes that mimicked natural seasonal variation, and UV lights (05.10d Outback max 10.0 UV fluorescent tube; Ultimate Reptile Suppliers, South Australia) were arranged above each enclosure (30 cm), emitting UVA and UVB radiation (10 % at 30 cm). Additionally, a heat lamp was suspended in each enclosure to generate a thermal gradient, and allow animals to attain their preferred body temperatures (approx. 36 °C; Walker unpublished data). Lizards were misted with water and fed live crickets three times per week.

### Captive breeding

Seventeen females successfully mated and produced offspring with males representative of different morphs, within and between two breeding seasons (September - January) in 2012–2013 and 2013–2014. Our goal was to mate each female with two or more males of different colour morphs to partially account for maternal effects. Eggs were collected and weighed within twenty-four hours of being laid; each egg was then buried in damp vermiculite (60 mL dry vermiculite: 20 mL water) in individual sealed containers, and incubated at 28.5 °C until hatching (mean incubation time ± SE = 56.8 ± 0.23 days; range = 52 to 61 days; *n =* 106). This incubation temperature was chosen to maximise the number of male offspring produced, as this species exhibits temperature dependent sex determination with cooler temperatures (25 °C - 27 °C) producing more females and warmer temperatures (27.5 °C - 30 °C) favouring males [41]. In 2012–2013, this incubation temperature yielded 23 sons and 16 daughters (~59 % male-bias), and in 2013–2014 yielded 9 sons, 7 daughters and 3 unsexed individuals (~56 % male-bias), giving a total of 58 offspring.

### Confirmation of paternity

Maternity was known for all offspring; however, we could not be certain of paternity due to sperm storage in agamid lizards [42–44]. Females that showed no sign of becoming gravid were re-partnered with new males and the maximum period of sperm storage is not known, making it difficult to determine which male sired the offspring. Therefore, to confirm paternity we genotyped all adult lizards and offspring using microsatellite loci. We collected blood samples (50–100 μL) by venipuncture from the vena angularis (in the corner of the mouth). Red blood cells were harvested from whole blood by centrifugation and frozen at −20 °C until DNA extraction. Genomic DNA was extracted from red blood cells with proteinase-K and a GenCatch Blood and Tissue Genomic Mini-Prep Kit (Epoch Life Science, TX, USA). DNA samples were PCR amplified at eight microsatellite loci previously developed for *C. decresii* (Ctde03, Ctde05, Ctde08, Ctde12, Ctde21 and Ctde45; [38, 45]), or for the closely related *Ctenophorus pictus*([CP10 and CP11; [46]), using published PCR protocols [38]. Amplified PCR products were sent to Macrogen (Korea) for fragment visualisation, and fragment sizes were called using Peak Scanner ver. 1.0 (Applied Biosystems).

Prior to analysis, genotypes from 70 of the 71 wild-caught adults in the captive population were used to calculate Hardy-Weinberg equilibrium (HWE) and check for linkage disequilibrium between loci in Genepop version 4.2 [47]. We also used Cervus (version 3.0.7, [48]) to calculate allele frequencies, observed and expected heterozygosity, polymorphic information content and the frequency of null alleles for each locus for these individuals. We detected significant linkage disequilibrium between two loci (Ctde08 and Ctde45), and locus CP10 deviated from HWE and had a high proportion of null alleles. Consequently, we excluded Ctde08 and CP10 from the subsequent paternity analysis.

Paternity was assigned using the software package Cervus, which employs a maximum likelihood approach to determine the most-likely candidate sire based on the genotypes of parents and offspring (version 3.0.7, [48]). To do this, it estimates a likelihood-of-difference (LOD) score, which is the log ratio of the likelihood of one sire being the true parent over another. We first conducted a simulation of parentage analysis to calculate critical values of likelihood ratios and determine confidence of subsequent paternity assignments based on our data. We used the default parameters of 10,000 offspring and 1 % error rate [48], with 3 candidate fathers, 95 % of loci typed and a minimum of 5 loci typed. To determine whether offspring were sired by their putative fathers, we then analysed allele mismatches between putative fathers, mothers, and their offspring. Paternity for all captive bred offspring was known to be one of up to three candidates. We were therefore able to concentrate on most likely candidates in the population. Paternity was assigned when it matched the offspring at all loci, or mismatched at only one locus (to account for possible mutations). We assigned paternity to the candidate male with the highest LOD score, at a 95 % confidence level. Assigned fathers were manually verified across mother–offspring pairs and the clutch-mates through direct genotype comparison.

### Inducing and quantifying throat colouration

We implanted females with testosterone to induce expression of throat colour morphs [40]. This study confirmed that the testosterone-induced female morphs are discrete, objectively classifiable, and occur in similar frequencies as in males [40]. Testosterone elevation induced expression of orange more strongly than yellow in adult females but because yellow was also present in a subset of females prior to testosterone treatment, those females could be unambiguously classified as yellow or orange + yellow morph ([40]; Additional file 1: Figure S1]). However, quantitative colour expression in females was less than for males (approximately 2/3 expression of orange and ½ for yellow in females, compared to males). Nevertheless, throat colouration could be scored in the same way (morph assignment and proportion of orange, yellow or grey) for sires and dams.

Male *C. decresii* ordinarily develop throat colouration at sexual maturity (approx. 18 months of age). Therefore, for the offspring born in late 2013 to early 2104, we induced colour expression using testosterone. Given the very small body size of immature *C. decresii* (mean weight ± SE = 2.72 ± 0.17 g; SVL = 40.10 ± 0.75 mm; *n =* 24), silastic hormone implantation was inappropriate. We therefore administered testosterone to juveniles at 5–7 months of age via a daily application of 4.5uL of sesame oil mixed with crystalline testosterone powder (no. T1500, Sigma), at a dose of 0.025 g of testosterone per 1 mL of oil, to the dorsal surface every evening for 42 days (method adapted from [49]). Both sexes expressed all four colour morphs (Table 1; Additional file 1: Figure S2).

**Table 1:** Frequencies of colour morphs of parental individuals, and two cohorts of offspring, where G = Grey morph; O = Orange; OY = Orange + Yellow; and Y = Yellow morph. Proportion of total is indicated in brackets. Morph category was determined via segmentation analysis of standardised photographs taken at the peak of colour development

	G	O	OY	Y	Total
Parental					
Male	7 (0.3)	4 (0.17)	8 (0.36)	4 (0.17)	23
Female	2 (0.12)	5 (0.29)	6 (0.35)	4 (0.24)	17
Total	9	9	14	8	40
2012-2013 season offspring					
Male	5 (0.22)	7 (0.30)	2 (0.09)	9 (0.39)	23
Female	5 (0.31)	4 (0.25)	5 (0.31)	2 (0.13)	16
Total	10	11	7	11	39
2013-2014 season offspring					
Male	1 (0.11)	5 (0.56)	2 (0.22)	1 (0.11)	9
Female	1 (0.14)	2 (0.29)	3 (0.43)	1 (0.14)	7
Unsexed	0 (0)	3 (1)	0 (0)	0 (0)	3
Total	2	10	5	2	19

To objectively quantify throat colouration of males and of females and offspring at peak testosterone-induced colour expression, we took digital photographs using a Canon PowerShot SX1-IS camera (saved in RAW format), and calibrated the images with respect to radiance and light intensity (methods detailed in [40, 50]). We then performed a segmentation analysis on calibrated photos to quantify the proportion of yellow, orange and grey on the throat of each individual, as described in L Teasdale, M Stevens and D Stuart-Fox [37]. Briefly, this analysis standardised for brightness, and extracted proportions of the throat area based on the RGB values of each pixel according to user-defined threshold values. The threshold was set at 0.15 for both red and yellow, assigned based on analysis of a subset of the images across cohorts. The proportion of pixels with red values above the user defined threshold effectively distinguished orange colouration on the throat; therefore we refer to the proportion of orange throughout for simplicity. Image calibration and segmentation analyses were done using modified scripts written by John Endler and Martin Stevens, executed in MATLAB (The MathWorks, Inc., MA, USA). Individuals with <2 % orange pixels and <5 % yellow pixels in mature animals (<1 % for both orange and yellow in juveniles) were classified as not expressing orange or yellow colouration respectively (i.e. 0 expression) because cream throat colouration (particularly darker, more variable cream in adults) could sometimes result in orange or yellow values below these thresholds. The proportion of orange and yellow colouration above these thresholds was used to both assign morph category (based on the presence/absence of above-threshold values of orange and yellow) and to analyse the genetic basis of quantitative variation in orange and yellow. Percentages of orange and yellow were arcsin square-root transformed for all analyses. Colour morph frequencies for parental individuals and two cohorts of offspring are given in Table 1.

### Discrete models of morph inheritance

Using the 58 captive bred offspring (32 males, 23 females, 3 unsexed) from 25 clutches with 23 different known sires and 17 dams (Additional file 1: Table S1), we considered three likely models of Mendelian inheritance of the four discrete morphs: orange (O), yellow (Y), orange + yellow (OY) and grey (G) as well as sex linkage for each of the three models. Agamid lizards have a ZZ/ ZW sex determination system, with females the heterogametic sex [51, 52].

#### Model 1: one locus, four alleles

Under this model, each of the four alleles correspond to one of the four colour morphs.

#### Model 2: one locus, three alleles (O, Y, G) with co-dominant expression

Under this model both OO and OG individuals would be classified as phenotypically orange morph, YY and YG individuals would be phenotypically yellow, GG individuals would be phenotypically grey and OY individuals would be phenotypically orange + yellow. The amount of orange or yellow expressed on the throat of orange or yellow morph individuals would depend on whether they were homozygous or heterozygous.

#### Model 3: two loci (‘orange’ locus and ‘yellow’ locus), each with two alleles (O and o; Y and y respectively)

The two loci control the expression of orange and yellow respectively with presence of the dominant O and Y allele resulting in colour expression. Under this model both OOyy and Ooyy individuals would be classified as phenotypically orange, ooYy and ooYY individuals would be phenotypically yellow, ooyy individuals would be phenotypically grey and OoYy, OOYy, OoYY and OOYY individuals would be phenotypically orange + yellow.

Under both models 2 and 3, the genotype of orange or yellow phenotypes is unclear because zygosity cannot be determined *a priori*. For example, in the case of one locus with three co-dominant alleles, orange individuals could be OO or OG while yellow individuals could be either YY or YG. In the case of a two-locus model, orange individuals could either be Ooyy or OOyy (two recessive y alleles resulting in the absence of yellow) and yellow individuals could be ooYy or oo YY (two recessive o alleles resulting in the absence of orange). Due to co-dominant or dose-dependent expression, one might expect a bimodal distribution of the proportion of orange or yellow expressed on the throat corresponding to heterozygotes (OG and YG) and homozygotes (OO and YY) but this was not apparent in our dataset.

Therefore, we calculated the expected frequency of offspring phenotypes based on three different assumptions regarding the probability that orange or yellow phenotype parents were heterozygous or homozygous at the one or two loci (depending on the model). We assumed that phenotypically orange or yellow sires and dams have: 1) a 50:50 probability of being homozygous or heterozygous; 2) a probability of being homozygous or heterozygous based on estimated allele frequencies in the population (*N =* 56 adult males) as expected at Hardy-Weinberg Equilibrium (HWE) or 3) that homozygous individuals have above the mean observed proportion (based on the 56 adult males) of orange or yellow on their throats and heterozygous individuals have below the mean observed proportion of orange or yellow on their throats. Note that although we included a model based on HWE, Hardy-Weinberg segregation is unlikely to apply for functional traits subject to selection [53–55]. Based on the probability of inheriting O, Y or G alleles (Model 2) or O or o and Y or y alleles (Model 3) from each parent under the above assumptions, we calculated the expected frequency of offspring in each morph category and tested whether this differed significantly from the observed frequencies using Likelihood Ratio tests (see Additional file 2).

### Heritability of quantitative colour expression

In *C. decresii* the proportion of grey simply reflects the remainder of the throat without orange or yellow, hence we estimated additive genetic variance for the two non-grey elements, yellow and orange, via two approaches. First, we used the animal model approach ([56, 57]; see below), which generates a relationship matrix for all design individuals and is therefore able to incorporate and correctly assign information across the entire pedigree. However, given the limited size of our dataset, and because parameter estimation is achieved iteratively according to maximisation of (restricted) Likelihood, convergence proved problematic for all but the simplest candidate models. We therefore supplemented this approach with conventional parent-offspring regressions [58] which enabled exploration of additive genetic effects according to the sex of parents and offspring.

Orange + yellow (OY) individuals have throats with an orange centre surrounded by yellow [37; Figure 1c]. When a central orange patch is expressed in OY individuals, yellow may be expressed only in the area surrounding the central orange patch with no phenotypic “overlap” among the two colour components. Alternatively, orange may “overlay” yellow (i.e., obscure yellow in the central region where it would otherwise be visible). This is potentially important because it would directly affect how to assign yellow trait values in the OY morph. In the latter case the proportion of yellow would be more accurately represented as the sum of orange + yellow. We therefore explored the proportional coverage of each colour element in yellow (Y) versus OY morphs in the 56 adult males (Additional file 1: Figure S3). Although we acknowledge that additional environmental and/or genetic interactions may influence trait (co)expression, the data best support a scenario wherein orange overlays yellow. Specifically, yellow coverage in the Y morph is significantly greater than in the OY morph, yet near identical to the coverage of orange and yellow combined (Additional file 1: Figure S3). By comparison, orange coverage in the O morph is not significantly greater than in the OY morph (Additional file 1: Figure S3). We therefore parameterized yellow in OY morphs as the sum of orange and yellow.

Animal modelling was conducted using ASReml ([56, 59]; see below) and included offspring sex and cohort (2012–2013 and 2013–2014) as fixed factors. Generation (parent [F0] versus offspring [F1]) was included to account for the potential differences in developmental environments of each group. Models used all data (*N =* 102, including the 10 individuals born in captivity in 2011 from unknown wild fathers but excluding the three unsexed juveniles), which included individuals with zero values for phenotypic traits (i.e., below threshold expression of orange (<2 %) and yellow (<5 %) from segmentation analysis of images classed as zero expression). This allowed us to use as much pedigree information as possible, and zero values specified as missing values and estimated as a component of sparse model effects in the inverse relationship matrix as generated by ASReml [60].

In addition to the animal model, we used parent-offspring regression to estimate heritability for the proportion of orange and yellow only in individuals that expressed above threshold values of orange (≥2 %) and yellow (≥5 %) (i.e., zero values excluded). Specifically, we regressed the average proportions of throat colour components of parents (mid-parent) against those of their offspring (mid-offspring). We also performed dam-offspring and sire-offspring regressions to partition the maternal and paternal contribution (sire-offspring covariances are less likely to be influenced by maternal effects) and cross-correlations between orange and yellow expression in parents and their offspring; for example, expression of orange in sires and yellow in offspring, and yellow in sires with orange in offspring. Cross-correlations provide insight into whether the proportion of colour on the throat is likely to arise from a single genetic factor/ locus [58]. Last, we tested for differences in the heritability of orange and yellow using ANCOVA with offspring values as the dependent variable and parent values, parent sex (sire or dam) and their interaction as fixed factors. Clutch ID was included as a random factor. A significant interaction would indicate different slopes attributable to sires versus dams (i.e. different paternal and maternal contributions), implying that inheritance may be sex-linked [58].

## Results

### Confirmation of paternity

We analysed the genotypes of 70 unrelated wild-caught adults and found that six of the eight microsatellite loci were suitable for paternity assignment (Additional file 1: Table S2). Using our parameter settings, CERVUS assigned a sire to all 58 offspring for which we had candidate fathers, with 95 % confidence. We accepted assigned sires with mismatches of zero or one (to allow for mutations). These were confirmed as likely fathers based on breeding sire-dam pairings. We detected only one instance of sperm storage between clutches within years and none of sperm storage between years for the population, increasing our confidence in our paternal assignment.

### Discrete models of morph inheritance

In several instances we were able to exclude either autosomal or sex-linked inheritance under a given model based on clear incompatibility with observed parent and offspring phenotypes (see below). However, autosomal inheritance for models 2 and 3 were potentially compatible with the data. For these models, we statistically tested observed offspring phenotype frequencies against those expected given parental phenotypes.

#### Model 1: one locus, four alleles

Under this model, colour morph is controlled by a single locus with four alleles corresponding to the four morphs. We can reasonably exclude a model with a single autosomal locus with four alleles because it requires that at least one of the parents must have the same morph as the offspring. For the 58 offspring for which the phenotype is known for the offspring and both parents, 17 do not have the same morph as either of their parents (29 %). We can also exclude sex-linked inheritance for this model because females would need to express the same morph as their father (due to the species’ ZW sex chromosomes) and this is not the case for 17/23 (74 %) female offspring with known fathers.

#### Model 2: one locus, three co-dominant alleles

Under this model, colour morph is controlled by a single locus with three alleles, O, Y and G with co-dominant expression. We can exclude sex-linked inheritance for this model (i.e. locus on the Z chromosome) because sex-linked models predict that phenotypic frequencies in the heterogametic sex (females) represent allelic (rather than genotypic) frequencies. Females would inherit the Z with either the O or Y allele from their father, so could not be OY by definition under this model because OY is a heterozygote with co-dominant alleles. The fact that some females were classified as orange + yellow morph following testosterone implantation necessitates autosomal inheritance under Model 2.

Comparison of observed and expected offspring phenotypic frequencies under autosomal inheritance for Model 2 revealed that they differed significantly (Table 2), regardless of assumptions underlying assignment of parental genotypic probabilities (i.e. 50:50; HWE and threshold assumptions). Autosomal inheritance for Model 2, however, provided the second best fit to the observed data (see below for best fitting model) when we assigned parental genotypes assuming threshold colour expression (homozygote above mean, heterozygote below mean colour expression), which is consistent with co-dominance.

**Table 2:** Likelihood ratio tests for observed and expected morph frequencies under different models of autosomal inheritance

Model of inheritance	Probability of O or Y parents being homozygous or heterozygous	Morph	Expected freq.	Observed freq.	G df = 3	P
One locus, 3 alleles	Equal	O	18	21	13.47	0.004
		Y	21	13		
		OY	14.63	12		
		G	4.38	12		
	HWE	O	16.7	21	14.23	0.003
		Y	23.22	13		
		OY	13.32	12		
		G	4.76	12		
	Threshold	O	17.25	21	9.29	0.026
		Y	19.75	13		
		OY	15.25	12		
		G	5.75	12		
Two loci	Equal	O	13.38	21	**5.13**	**0.163**
		Y	15.63	13		
		OY	15.13	12		
		G	13.88	12		
	HWE	O	12.31	21	10.76	0.013
		Y	14.83	13		
		OY	21.35	12		
		G	9.51	12		
	Threshold	O	11	21	14.42	0.002
		Y	13.5	13		
		OY	23.5	12		
		G	10	12		

‘Equal’ refers to equal (50:50) probability; ‘HWE’ refers to estimated allele frequencies from the population assuming Hardy-Weinberg Equilibrium; ‘Threshold’ refers to homo- or heterozygoisty assigned based on a threshold for which we used the mean proportion of throat colour (56 adult males). Bold indicates that observed frequencies do not differ significantly from those expected under that model

#### Model 3: two loci, each with two alleles

Under this model, there are two loci - an ‘orange’ locus and a ‘yellow’ locus. In the case of sex-linkage for this model, a grey father must produce grey daughters, a yellow father could not produce daughters that express orange and an orange father could not produce daughters that express yellow. Of the 4 female offspring with a grey father, 3 were non-grey daughters; of the 7 female offspring with a yellow father, 3 were orange or orange + yellow and of the 3 female offspring with an orange father, one was yellow, totalling 7/14 (50 %) of offspring phenotypes that do not fit sex-linked inheritance under Model 3.

By contrast, observed offspring phenotypic frequencies did not differ significantly from those expected under autosomal inheritance for Model 3, but only under one of the three assumptions regarding parental genotypic probabilities; namely, when we assumed a 50:50 probability of phenotypically orange or yellow parents being homozygous or heterozygous for the orange and yellow locus respectively (Table 2). Observed and expected phenotypic frequencies differed significantly when allele frequencies were assumed to be in HWE or estimated based on a threshold of colour expression (Table 2).

### Heritability of quantitative colour expression

Animal model analysis including all individuals (i.e. zero values estimated as a sparse term) indicated high and significantly > 0 heritability for the proportion of orange (0.84; 95 % CI 0.55 – 1.12; Table 3) and the proportion of yellow (0.67; 95 % CI 0.30 – 1.04; Table 3).

**Table 3:** Results of animal models estimating heritability for the proportion of orange and yellow throat colouration (design size, *N =* 102; see methods for details)

Response variable	Animal variance	Residual variance	Phenotypic variance	Heritability h^2^ ± SE	95 % confidence limits
Proportion orange	10.80	2.11	12.91	0.84 ± 0.14	0.55 –1.12
Proportion yellow	8.20	4.05	12.25	0.67 ± 0.19	0.30 –1.04

**Fig. 2:**
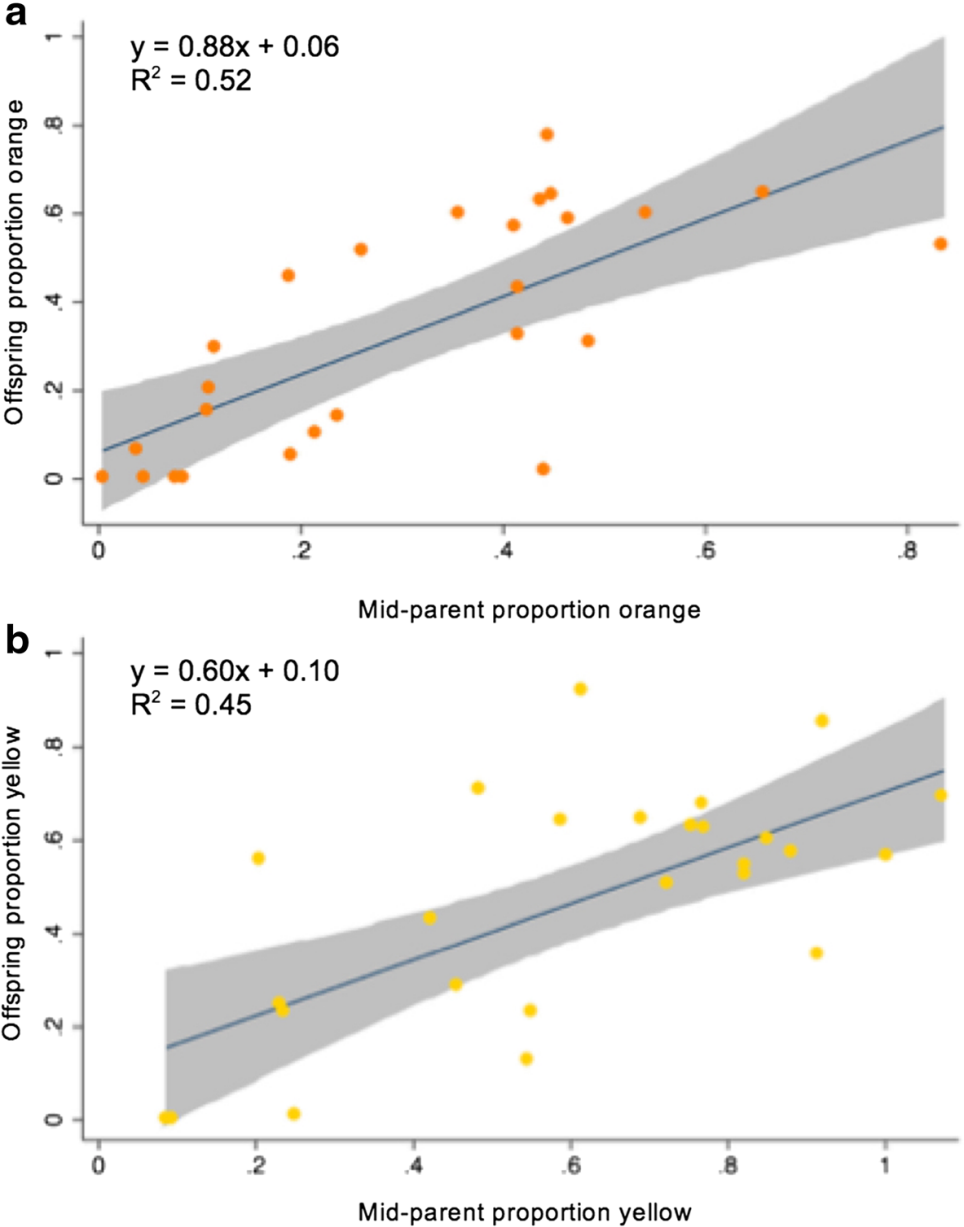
Parent-offspring regressions of the proportion orange and yellow components of throat colouration. Heritability estimate (h^2^) is given by the slope of the regression: A) orange h^2^ = 0.88, *p <* 0.001; B) yellow h^2^ = 0.60, *p =* 0.0002. Grey shading shows 95 % confidence intervals around the slope

Mid-parent-offspring regressions for individuals expressing orange or yellow (i.e. zero values excluded) indicated high heritability for each component of throat colour (Fig. 2). Heritabilities for the expression of both orange and yellow were significant and comparable to those estimated from the animal model, being higher for the proportion of orange (h^2^ = 0.88 ± 0.17, *p =* <0.001) than yellow (h^2^ = 0.60 ± 0.13, *p =* 0.002).

**Fig. 3:**
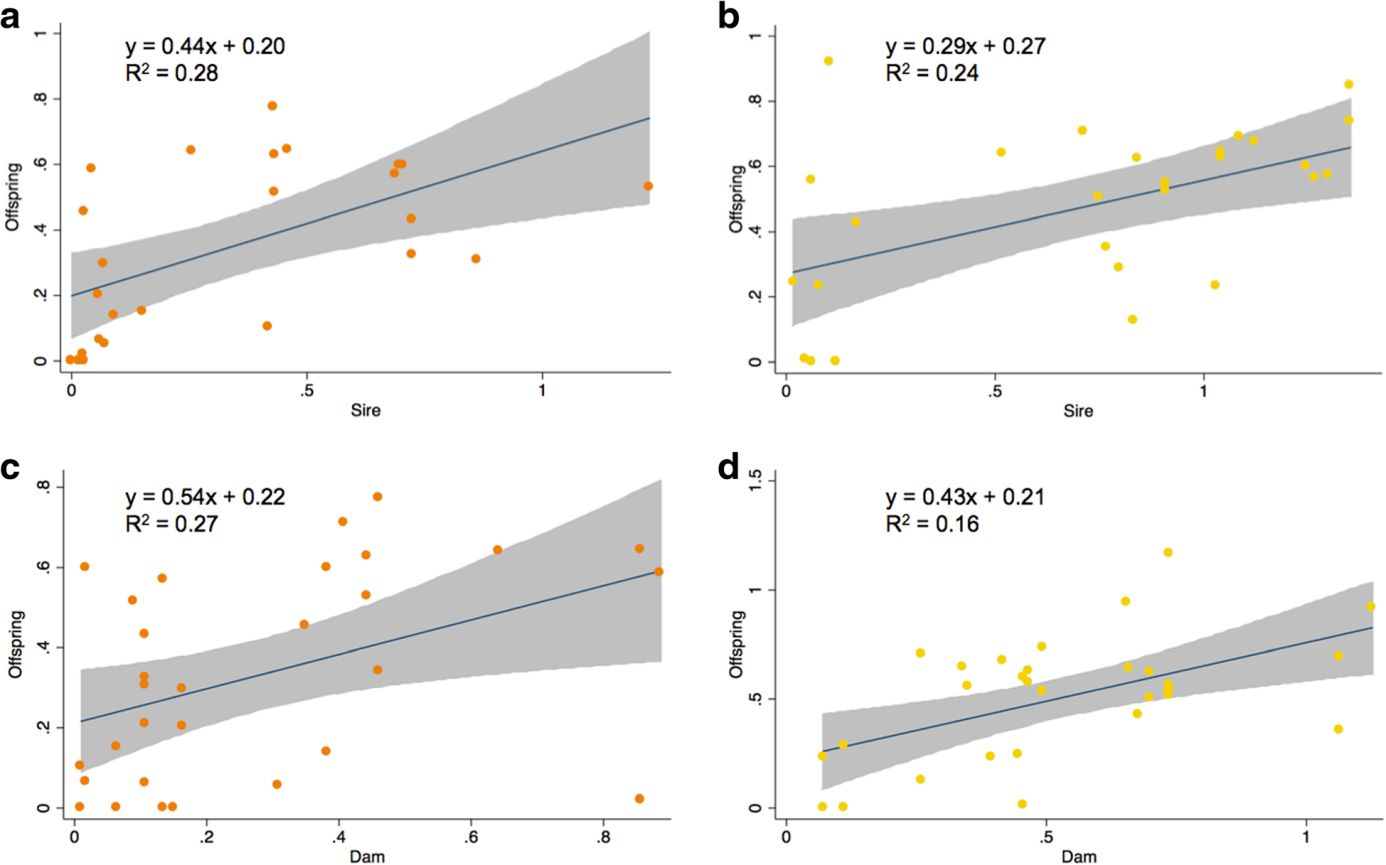
Sire-offspring regressions (**a**, **b**) and dam-offspring regressions (**c**, **d**) of proportion of orange (**a**, **c**) and yellow (**b**, **d**) components of throat colouration. Heritability (h^2^) is given by the slope of the regression. Grey shading shows 95 % confidence intervals around the slope

Despite the limited sample size, the proportion of orange was significantly heritable in sire-offspring regressions (h^2^ = 0.44 ± 0.13, *p =* 0.003; Fig. 3a), as was the regression for the proportion of yellow (h^2^ = 0.29 ± 0.09, *p =* 0.005; Fig. 3b; Table 4). Similarly, heritability for the proportion of both orange and yellow was significant for dam-offspring regressions (h^2^ = 0.43 ± 0.17, *p =* 0.016, and h^2^ = 0.54 ± 0.16, *p =* 0.002 for orange and yellow, respectively; Fig. 3c, d; Table 4). Furthermore, there was no significant difference in slopes between sire-offspring and dam-offspring relationships for orange (ANCOVA: parent colour x parent sex interaction; F_1,50_ = 0.00; *p =* 0.97) and a marginally significant difference in slopes for yellow (F_1,50_ = 5.45; *p =* 0.024). This implies that maternal and paternal contributions were of comparable magnitude for orange but not yellow. Lastly, cross-correlations of orange and yellow were moderate but statistically significant (and positive) for all except yellow dam versus orange offspring (Table 4).

**Table 4:** Coefficient estimates from sire-offspring and dam-offspring regressions and cross correlation (reciprocal phenotypic regressions) of the proportion of orange and yellow components of throat colouration. Morph category was determined via segmentation analysis of digital photographs

Offspring	Sire	Dam	Slope ± SE	R^2^	P
Orange	Orange	-	0.44 ± 0.13	0.28	0.003*
Orange	-	Orange	0.43 ± 0.17	0.16	0.016*
Orange	Yellow	-	0.31 ± 0.10	0.26	0.004*
Orange	-	Yellow	0.25 ± 0.17	0.04	0.15
Yellow	Yellow	-	0.29 ± 0.09	0.24	0.005*
Yellow	-	Yellow	0.54 ± 0.16	0.27	0.002*
Yellow	Orange	-	0.35 ± 0.14	0.17	0.018*
Yellow	-	Orange	0.42 ± 0.19	0.12	0.033*

* denotes significance

## Discussion

Our results confirm that in *Ctenophorus decresii*, like many other colour polymorphic taxa (reviewed in [6]), discrete colour morphs are likely to be controlled by few genetic factors that segregate in a Mendelian fashion. Of the three models of inheritance we considered, a model positing two autosomal loci controlling the presence/absence of orange and yellow respectively provided the best fit. Quantitative expression of the proportion of orange and yellow within colour morphs indicated heritability estimates for orange (0.84; 95 % CI 0.55 – 1.12) being higher than for yellow (0.67; 95 % CI 0.30 – 1.04). Furthermore, cross-correlations (indicative of genetic covariance) between orange and yellow were significant and positive for all except yellow dam versus orange offspring, suggesting that their quantitative expression may be controlled by few or tightly linked loci. Thus, our study supports theoretical predictions that discrete colour morphs associated with distinct behavioural strategies should be governed by few genes of major effect (e.g. a supergene, [13, 61]) and provides evidence for heritability of quantitative colour expression among individuals with discrete colour morphs.

Colour polymorphism in a wide range of taxa is controlled by few or tightly linked loci, which may have a pleitropic effect on correlated traits (especially when they involve regulatory genes) or form part of a ‘supergene’ that limits effective recombination between constituent loci to form a single segregating unit [21]. For instance, regulatory genes have been implicated in control of the colour polymorphism in the swallowtail butterfly *Papilio polytes* (transcription factor doublesex (dsx), [25, 62]) and Lake Malawi cichlids (cis-regulatory mutation in the Pax7 gene; [23]); while the plumage colour polymorphism in white-throated sparrows (*Zonotrichia albicollis*) and ruffs (*Philomachus pugnax*) is associated with a supergene [9, 63]. Our data are similarly consistent with Mendelian inheritance of few alleles at limited loci. However, interactions among loci or between genes can make substantial contributions to trait variation and even covariation [64] so we cannot rule out more complex models involving multiple genes or epistatic interactions [13, 65, 66].

Based on our data, we can reject sex-linked inheritance of the potential for discrete polymorphism based on observed offspring phenotype frequencies. Furthermore, we can reject sex-linked inheritance of genes involved in quantitative expression of orange colouration, as there was no significant difference in the slopes of sire-offspring and dam-offspring regressions. The significant difference in slopes for sire-offspring and dam-offspring regressions for yellow colouration may be an artefact of limited testosterone-induced expression of yellow in adult females compared to juveniles, rather than indicative of sex-linkage. Even in species with sex-limited polymorphism (where the colour polymorphism is expressed only in in one sex), autosomal inheritance appears to be substantially more common than sex-linked inheritance (e.g. ruffs [8, 9], side-blotched lizard [12], damselflies [67], butterflies [68]), although there are exceptions (e.g. Gouldian finch [1] *Ficedula* flycatchers [69]). If conspicuous colouration benefits one sex (usually males) but disadvantages the other (usually females, which gain greater fitness from being cryptic), then autosomally inherited colour traits will be under sexually antagonistic selection. The resulting genetic conflict may be resolved through tight linkage with sex-determining genes (e.g. Lake Malawi cichlids; [23]), or alternatively, through the action of ‘modifier’ genes (e.g. endocrine-associated) that limit colour expression to one sex during development [23]. The latter case is likely in *C. decresii* as well as other species such as ruffs (*Philomachus pugnax*), side-blotched lizards (*Uta stansburiana*), painted dragons (*Ctenophorus pictus*), in which testosterone mediates colour morph expression [40, 70, 71]. Indeed testosterone-induced colour expression in females and juveniles further indicates that sex-specific endocrine cascades during development, rather than linkage with sex-determining genes, control the expression of throat colouration.

Lizards show remarkable convergence in male throat or ventral colour polymorphism (with orange, yellow and white/grey or blue morphs), which appears to have evolved independently in several families, including iguanids, lacertids and agamids [22, 72–77]. This is intriguing because it raises the possibility of a common underlying genetic mechanism. However, until now, there have been only two colour polymorphic lizard species for which the probable mode of inheritance for the colour polymorphism has been estimated: the side-blotched lizard, *Uta stansburiana* [12], and the painted dragon, *Ctenophorus pictus* [78]. Male throat colour polymorphism in the side-blotched lizard appears to be controlled by a single locus with three, co-dominant alleles (*b, o* and *y*), with both alleles expressed in heterozygotes [79]. Homozygous males have solid throat colours; blue (*bb*), orange (*oo*), or yellow (*yy*). Due to co-dominance, heterozygotes have intermediate phenotypes; blue-yellow (*by*) have alternating blue and yellow stripes, blue-orange (*bo*) have blue and orange stripes on the throat with light orange flanks, yellow-orange (*yo*) have yellow throats with blue stripes and pale orange flanks [79]. The colour morphs correspond to reproductive strategies with ‘orange’ oo, oy and ob males having an ‘ultra-dominant’ strategy (large territories), ‘yellow’ yy and yb males having a sneaker strategy and ‘blue’ bb males having a mate-guarding strategy [11, 22, 79]. In contrast to the side blotched lizard, in the painted dragon, data was inconclusive regarding the mode of inheritance for males’ yellow, orange or red head colouration. For *C. pictus*, a single locus three-allele model could not be definitively rejected, though it was not strongly supported, and there was no support for polygenic inheritance or a simple two-allele co-dominance model. However, neither of these studies formally considered models of inheritance with more than one locus.

In *C. decresii*, the only model of inheritance consistent with observed offspring phenotype frequencies was one with two loci (and ‘orange’ locus and a ‘yellow’) locus, each with two alleles and with the allele coding for orange or yellow expression respectively being dominant (‘two-locus model’). However, we could not reliably score genotype from phenotype for the models of inheritance we tested formally (in contrast to the side-blotched lizard, for which genotype can be scored from phenotype due to co-dominant allelic expression). Therefore, we estimated offspring phenotype frequencies according to three different assumptions regarding parental genotypic frequencies. Our data provided the strongest fit with the two-locus model when we assumed a 50:50 probability of sires and dams of a given phenotype being either heterozygous or homozygous. However, a model with a single autosomal locus and three co-dominant alleles (analogous to the side-blotched lizard), fit the observed offspring phenotype frequencies almost as well as the best model (when we assumed threshold colour expression, which is consistent with co-dominance). Additional data would be necessary to further test among these competing models.

In addition to suggesting Mendelian inheritance of the discrete polymorphism, our results show that the quantitative expression of orange and yellow is highly heritable, with heritability of orange expression greater than that of yellow. This is consistent with the mechanism of colour production in *C. decresii*. In this species, yellow colouration is produced by carotenoids (e.g. β-carotene, 3’-dehydrolutein and lutein/zeaxanthin), while orange is produced by the same carotenoids with the addition of the red pteridine, drosopterin, as well as other colourless pteridines (C.A. McLean, A. Lutz, K. Rankin and D. Stuart-Fox, unpublished data). This is similar to most other agamid and iguanid lizards studied to date, whereby yellow is generated primarily by carotenoids and orange is generated by the combination of carotenoids and drosopterin (e.g. [80, 81]). Carotenoids and pteridines are the two primary classes of pigment generating yellow to red colouration in reptiles but they have very different chemical structures and physiological roles and are produced in different ways [82]. Animals acquire carotenoids exclusively from the diet; therefore, carotenoid-based ornaments are widely viewed as condition-dependent, with colour expression depending on both environmental availability and allocation trade-offs (e.g. to ornamentation vs immune function; [83, 84]). By contrast, coloured pteridines comprise a subset of chemical compounds (sepiapterin, drosopterin and their derivatives) synthesised within specialised organelles (pterinosomes) within the pigment cells (xanthophores) from abundant precursors presumed to be non-limiting [82, 85]. Therefore, in sharp contrast to carotenoids, environmental influences on the expression of pteridine-based colours are thought to be minimal [86]. In guppies, variation among populations in drosopterin production is largely genetic and compensates for environmental variation in carotenoid availability [86, 87]. Thus, higher heritability of orange colouration in *C. decresii* is consistent with genetic control of drosopterin production contributing to the generation of orange.

## Conclusions

Based on our results, we hypothesise hierarchical genetic control of colour expression in *C. decresii*. Two independently segregating loci may determine whether or not orange or yellow is expressed (i.e. pigment is produced or transported to the skin). If either (or both) of these ‘genes’ is ‘switched on,’ a physiological cascade influenced by multiple genes ensues (and potentially environmental effects and interactions), such that the ultimate extent of colour expression behaves in the manner of a quantitative trait. Furthermore, the potential genetic covariation between yellow and orange suggests that loci influencing quantitative expression influence both yellow and orange. The greater environmental component of variation in yellow (i.e. lower heritability) than orange is consistent with different mechanisms of colour production, although it is also possible that this reflects greater variation in testosterone-induced yellow colouration in females and juveniles. The relationship between genetic factors governing discrete morphs, mechanisms of colour production and quantitative colour expression is likely to be complex, and would require extensive pedigree data, linkage mapping and genomic analyses to fully elucidate. The evidence we present for heritability of both the discrete colour polymorphism and quantitative colour expression in *C. decresii* provides an important first step towards this endeavour.

## Additional files

Additional file 1:
**Figures S1-S2.** Show effect of testosterone application on throat colour expression of mature females and juveniles, respectively. **Figure S3.** Shows proportional coverage of each colour element in yellow (Y) versus OY morphs in the 56 adult males. **Table S1.** Presents pedigree of fifty-eight offspring across two breeding seasons used to assess models for inheritance. **Table S2.** Presents characteristics of microsatellite loci based on 70 unrelated wild-caught adults. (PDF 9495 kb)
Additional file 2: Analysis of expected and observed allele frequencies under proposed models of inheritance. (XLSX 65 kb)
