# Supplementary material for: The genetic basis of discrete and quantitative colour variation in the polymorphic lizard, Ctenophorus decresii
Source: BMC Evol Biol. 2016 Sep 6;16:179. doi: 10.1186/s12862-016-0757-2 (PMC5012029; doi:10.1186/s12862-016-0757-2)
Supplement: Additional file 1: — Figures S1-S2. Show effect of testosterone application on throat colour expression of mature females and juveniles, respectively. Figure S3. Shows proportional coverage of each colour element in yellow (Y) versus OY morphs in the 56 adult males. Table S1. Presents pedigree of fifty-eight offspring across two breeding seasons used to assess models for inheritance. Table S2. Presents characteristics of microsatellite loci based on 70 unrelated wild-caught adults. (PDF 9495 kb) [file 12862_2016_757_MOESM1_ESM.pdf]

SUPPLEMENTARY INFORMATION

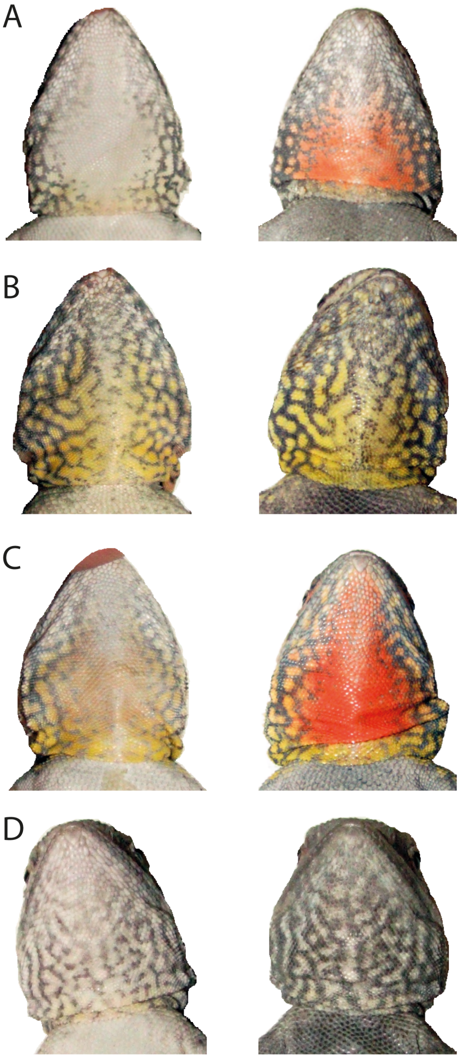

Figure S1. Effect of testosterone implantation on mature female throat colour expression for each morph. Pre-testosterone implantation (left) versus same lizard at peak of morph expression (right): A) orange, B) yellow, C) orange+yellow, D) grey.

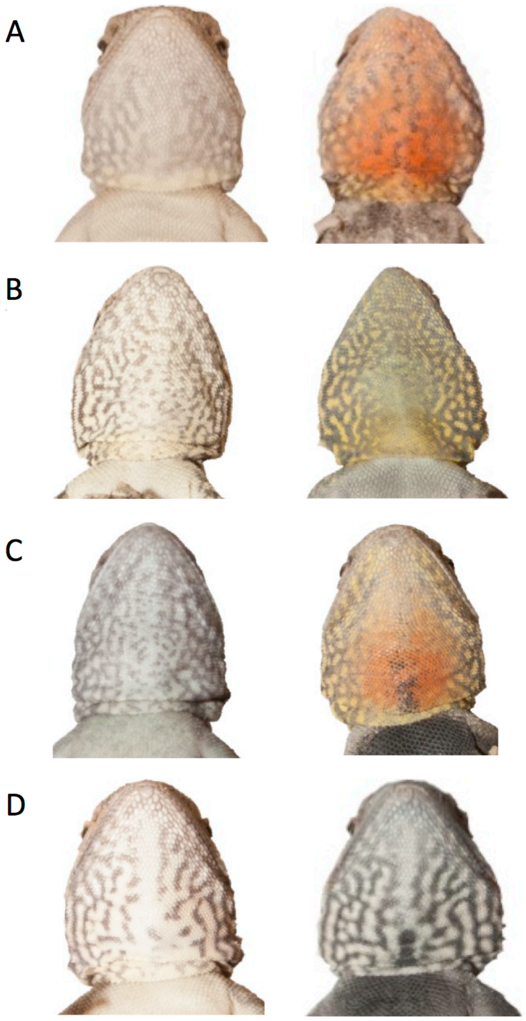

Figure S2. Effect of testosterone-oil rub on juvenile throat colour expression for each morph. Pre-testosterone application (left) versus same lizard at peak of morph expression (right): A) orange, B) yellow, C) orange+yellow, D) grey.

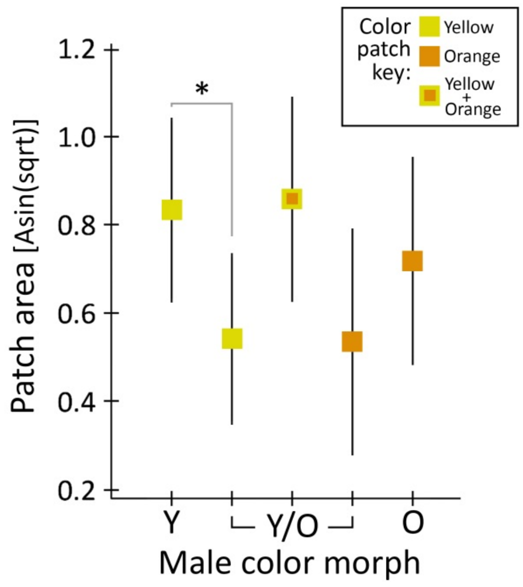

Figure S3. Mean (± 1 S.D.) coverage of yellow and orange throat colouration across wild-sampled male *Ctenophorus decresii* morphs categorized as either yellow (Y; N = 10), orange (O; N = 11) or yellow/orange (Y/O; N = 22). Yellow coverage is significantly greater in the Y versus Y/O morph (*$F_{1,30}$ = 15.3, $P < 0.001$), but indistinguishable from the sum of yellow + orange in the Y/O morph ($F_{1,30}$ = 0.04, $P = 0.85$). By comparison, orange coverage in the Y/O morph is does not differ significantly from that in the pure O morph ($F_{1,31}$ = 3.98, $P = 0.06$). The Y-axis is scaled as ArcSin(square-root)-transformed proportional coverage of colour patch elements.

Table S1. Pedigree of fifty-eight offspring across two breeding seasons used to assess models for inheritance; 32 males (M) : 23 females (F) : 3 unsexed (Juv) from 25 clutches with 23 different sires, and 17 dams. Morph types determined via segmentation analysis of digital photographs.

Offspring ID	Hatching date	Sex	Morph	Dam ID	Morph	Sire ID	Morph
203.1	19/12/12	M	Y	203	OY	258	Y
203.2	19/12/12	F	Y	203	OY	258	Y
203.4	19/12/12	F	OY	203	OY	258	Y
203.5	23/01/13	M	O	203	OY	257	O
203.6	23/01/13	M	O	203	OY	257	O
205.4	24/01/13	M	G	205	G	269	G
205.6	4/11/13	M	G	205	G	208	OY
205.7	5/11/13	F	OY	205	G	208	OY
206.11	7/11/13	M	Y	206	Y	213	O
206.3	14/12/12	M	G	206	Y	224	OY
206.4	14/12/12	M	OY	206	Y	224	OY
206.5	14/12/12	M	Y	206	Y	224	OY
206.6	14/12/12	F	Y	206	Y	224	OY
206.7	8/11/13	F	G	206	Y	213	O
206.8	5/11/13	M	O	206	Y	213	O
206.9	6/11/13	M	OY	206	Y	213	O
209.2	19/01/13	M	Y	209	O	262	Y
209.5	13/11/13	Juv	O	209	O	210	OY
209.6	12/11/13	Juv	O	209	O	210	OY
209.8	13/11/13	M	O	209	O	210	OY
211.1	7/01/13	F	OY	211	OY	253	Y
211.3	7/01/13	M	OY	211	OY	253	Y
211.4	7/01/13	F	OY	211	OY	253	Y
211.5	7/01/13	M	Y	211	OY	253	Y
211.6	7/01/13	M	Y	211	OY	253	Y
218.3	16/01/14	F	O	218	O	230	OY
219.3	11/11/13	F	OY	219	O	269	G

220.2	7/01/13	F	OY	220	Y	214	OY
220.3	9/01/13	F	O	220	Y	214	OY
221.1	9/11/12	M	O	221	OY	222	G
221.2	9/11/12	M	G	221	OY	222	G
221.4	9/11/12	F	O	221	OY	222	G
226.1	18/12/12	M	Y	226	OY	240	G
227.4	4/11/13	M	OY	227	OY	263	G
232.1	17/12/12	F	O	232	O	256	O
232.3	17/12/12	M	O	232	O	256	O
232.6	24/11/13	Juv	O	232	O	214	OY
232.8	25/11/13	M	O	232	O	214	OY
238.11	17/12/12	M	O	238	O	245	OY
238.12	17/12/12	F	OY	238	O	245	OY
238.8	17/12/12	M	O	238	O	245	OY
238.9	17/12/12	M	O	238	O	245	OY
242.1	14/12/12	F	O	242	G	264	OY
242.2	14/12/12	F	G	242	G	264	OY
242.3	14/12/12	M	Y	242	G	264	OY
242.4	14/12/12	M	Y	242	G	264	OY
242.5	14/01/13	M	G	242	G	217	G
242.6	14/01/13	F	G	242	G	217	G
251.1	4/01/13	F	G	251	Y	267	Y
251.2	4/01/13	F	G	251	Y	267	Y
251.3	4/01/13	M	Y	251	Y	267	Y
251.4	4/01/13	F	G	251	Y	267	Y
251.5	3/01/14	F	OY	251	Y	238.4	O
251.6	5/01/14	M	O	251	Y	238.4	O
251.7	6/01/14	M	O	251	Y	238.4	O
261.3	5/01/13	M	G	261	Y	248	G
261.6	8/11/13	F	O	261	Y	234	OY
206.1.1	18/11/13	F	Y	206.1	OY	238.3	G

Table S2. Characteristics of microsatellite loci based on 70 unrelated wild-caught adults: Number of alleles (k), number of individuals typed (N), observed (H_o) and expected (H_e) heterozygosity, p-value from exact test of Hardy-Weinberg equilibrium (HWE) and exclusion probabilities of microsatellite loci used for paternity confirmation. Polymorphic information content (PIC) is a measure of informativeness related to expected heterozygosity of the loci. Probability of maternal exclusion is the probability that a randomly selected adult will not match the offspring at a locus (when neither parent is known), and probability of paternal exclusion is the probability of excluding a randomly selected male as the sire, given the genotype of the mother and offspring.

Locus	k	N	H_o	H_e	PIC	HWE	Prob. of maternal exclusion	Prob. of paternal exclusion	Null allele frequency
Ctde3	20	142	0.894	0.938	0.931	0.212	0.235	0.133	0.0214
Ctde5	19	147	0.918	0.912	0.903	0.395	0.308	0.182	-0.0049
Ctde8	17	144	0.854	0.852	0.832	0.286	0.463	0.299	-0.0032
Ctde12	20	148	0.878	0.911	0.901	0.341	0.31	0.184	0.0178
Ctde21	18	147	0.816	0.839	0.821	0.726	0.471	0.306	0.0116
Ctde45	11	148	0.804	0.806	0.774	0.371	0.567	0.39	0
CP11	6	149	0.443	0.451	0.423	0.079	0.89	0.737	0.0009
